# Influence of Epoxy Resin Curing Kinetics on the Mechanical Properties of Carbon Fiber Composites

**DOI:** 10.3390/polym14061100

**Published:** 2022-03-09

**Authors:** Isidro Cruz-Cruz, Claudia A. Ramírez-Herrera, Oscar Martínez-Romero, Santos Armando Castillo-Márquez, Isaac H. Jiménez-Cedeño, Daniel Olvera-Trejo, Alex Elías-Zúñiga

**Affiliations:** 1 Mechanical Engineering and Advanced Materials Department, School of Engineering and Science, Tecnologico de Monterrey, Ave. Eugenio Garza Sada 2501 Sur, Monterrey, N. L. 64849, Mexico; isidro.cruz@tec.mx (I.C.-C.); claudia.ramirez@ciqa.edu.mx (C.A.R.-H.); daniel.olvera.trejo@tec.mx (D.O.-T.); 2 Vertiv, Ave. Luis Guadalupe Fernandez 3502 Parque Industrial FINSA Santa Catarina, Santa Catarina, N. L. 66380, Mexico; santoscastillo.aim@gmail.com (S.A.C.-M.); isaac.jimenez@vertiv.com (I.H.J.-C.)

**Keywords:** epoxy resins, light-weighting components, differential scanning calorimetry, kinetic parameters, carbon-fiber-reinforced composites, mechanical properties, aerospace applications

## Abstract

In this study, the kinetic parameters belonging to the cross-linking process of a modified epoxy resin, Aerotuf 275-34™, were investigated. Resin curing kinetics are crucial to understanding the structure–property–processing relationship for manufacturing high-performance carbon-fiber-reinforced polymer composites (CFRPCs). The parameters were obtained using differential scanning calorimetry (DSC) measurements and the Flynn–Wall–Ozawa, Kissinger, Borchardt–Daniels, and Friedman approaches. The DSC thermograms show two exothermic peaks that were deconvoluted as two separate reactions that follow autocatalytic models. Furthermore, the mechanical properties of produced carbon fiber/Aerotuf 275-34™ laminates using thermosetting polymers such as epoxies, phenolics, and cyanate esters were evaluated as a function of the conversion degree, and a close correlation was found between the degree of curing and the ultimate tensile strength (UTS). We found that when the composite material is cured at 160 °C for 15 min, it reaches a conversion degree of 0.97 and a UTS value that accounts for 95% of the maximum value obtained at 200 °C (180 MPa). Thus, the application of such processing conditions could be enough to achieve good mechanical properties of the composite laminates. These results suggest the possibility for the development of strategies towards manufacturing high-performance materials based on the modified epoxy resin (Aerotuf 275-34™) through the curing process.

## 1. Introduction

The automotive and aeronautic industries are developing efficient means of transport considering aspects such as energy consumption, environmental impact, weight, and cost. One possible way to achieve good results focuses on developing advanced materials considering successful cases reported with metal alloys and composite materials based on ceramics reinforced with carbon nanotubes, graphene, or carbon fibers [1,2,3,4,5,6,7,8,9,10,11,12]. Some properties of interest include fatigue resistance, durability, tensile strength, and corrosion resistance in submarine and salt-rich environments where the replacement of metals is desirable [13,14,15]; low energy power consumption during the manufacturing process; and relatively fast processing and self-healing capability on fracture [16]. Among several alternatives, carbon-fiber-reinforced polymer composites (CFRPCs) have emerged as an excellent choice, meeting most of these characteristics because of their manufacturability and superior properties [1,2,3,11,12]. For instance, in the aerospace industry, there is a continuous search for lightweight materials with adequate mechanical properties that can be manufactured by simple processes. In this sense, Lamborgini [17] developed carbon fiber/thermoset resin composites that can be used for producing the inner monocoque and suspension control arms of the *Sesto Elemento*. This technology is based on chopped carbon fiber prepregs which are randomly distributed into a mold for forging at determined pressure and temperature, whereas the curing process is in progress. The use of temperature allows the resin to flow, and the applied pressure facilitates the consolidation of the composite material, while voids are suppressed. This process has the benefit of reducing the manufacturing time and cost in comparison to others previously used [17].

Typically, CFRPCs have been developed by using thermosetting resins such as epoxies, phenolics, and cyanate esters; however, the right choice depends on their compatibility with the carbon fibers, their processing conditions (curing time, temperature, pressure, among others), as well as their physicochemical properties. For aerospace applications, the thermosetting resin must fulfil special requirements such as ease of manufacturability, cost effectiveness, possibility of mass production, durability, light weight, high-performance mechanical properties, and good resistance properties for fire, smoke, and toxicity [18]. In this context, sophisticated three-dimensional designs of aeronautical components with composite materials are strongly dependent on the resin properties, and epoxy resins are among the best options. Epoxy resins are characterized by the low viscosity of their formulations, good chemical and corrosion resistance, high durability, good thermomechanical properties, and, due to epoxy resin bonding to almost all materials, remarkable adhesion properties [19,20]. Moreover, the curing process of epoxy resins can be enhanced using several curing agents such as amines, polyamides, or imidazoles [20]. The most common thermoset resins used for aeronautics applications are the epoxy resins based on bisphenol A [21]. This resin is a common compound in the plastic industry that is used as an intermediate in the binding, plasticizing, and hardening processes, as well as in the manufacture of some forms of epoxy resins. Additionally, it is used as an additive for flame-retardant materials, as a stabilizer in PVC, and as an antioxidant agent in brake fluid, rubber, and plastics. In the case of polar polyamide matrix, bisphenol A is tightly bound. These properties justify the use of bisphenol A as a component in epoxy resins for aerospace applications [22]. Epoxy resins based on bisphenol A are an excellent alternative due to their outstanding advantages such as high strength and stiffness, toughness, adhesive properties, high thermal and chemical resistance [21,23,24], as well as their specific flame-retardant properties required in the aerospace industry to meet tough flame–smoke–toxicity (FST) requirements.

Safran Group developed a modified epoxy thermoset resin with the trade name Aerotuf 275-34™. To control the exotherm and cure cycle of Aerotuf 275-34™, the curing kinetic parameters must be determined to establish the structure–property–processing relationships for manufacturing high-performance CFRPCs from modified epoxy thermoset resin. Diverse analytic techniques such as Fourier-transform infrared (FTIR) spectroscopy [25,26,27,28], differential scanning calorimetry (DSC) [21,26,27,29,30], Raman spectroscopy [31,32], nuclear magnetic resonance (NMR) [33,34], and rheological analysis, e.g., dynamo-mechanical analysis (DMA) [13,35], have been employed to monitor the extent of the curing reaction of epoxy resins. From these techniques, DSC is one of the most extensively used due to its suitability and simplicity in determining kinetic parameters [21,26,27,29,30]. Although the precise chemical structure of Aerotuf 275-34™ is unknown, previous studies indicate that some functional groups are related to amine, imide, and maleimide units, and therefore the resin could be identified as a bisphenol A resin derivative [28]. Independently of the chemical nature, kinetics parameters can be obtained in a relatively simple manner through the DSC technique. Moreover, the validation of kinetics studies of resin in the processing of composites and their influence on the mechanical properties of final parts is still scarce [27,35].

Since an exhaustive understanding of the curing process is important for the development of aeronautical components, this work focused on studying the curing kinetics of Aerotuf 275-34™ using the DSC technique. We also investigated the relationship between the resin curing process and the mechanical properties when the thermoset resin is used in the production of lightweight [±45] woven carbon fiber fabric (WCFF)/Aerotuf 275-34^TM^ composite laminates that were produced by compression molding.

## 2. Materials and Methods

### 2.1. Materials

Aerotuf 275-34™ epoxy resin (here simply named as Aerotuf) was used in this study for the kinetics analysis and as an impregnation resin. The [±45°] WCFF/Aerotuf prepregs (60% fiber content and 40% Aerotuf resin, by weight) were used to produce composite laminates. Prior to use, and following the supplier specifications, Aerotuf resin and [±45°] WCFF/Aerotuf prepregs were stored at −20 °C. As a mold release agent Ease Release 200 (Mann Release Technologies, Inc., Macungie, PA, USA) was used.

### 2.2. Composite Laminates

**Figure 1:**
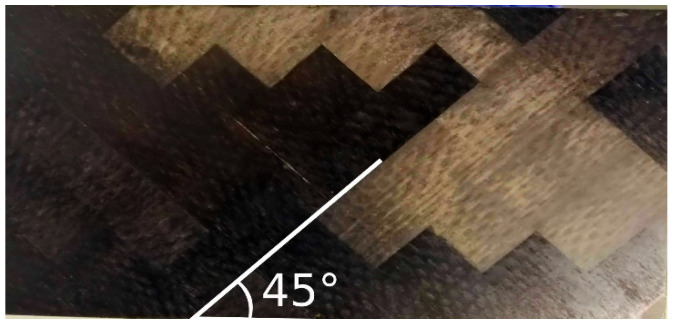
Composite laminates (250 × 135 × 2.5 mm^3^ in size) at the end of the curing process and after removing from the stainless-steel mold.

Carbon-fiber-reinforced composite laminates were manufactured from [±45°] WCFF/Aerotuf prepregs. The notation [±45°] stands for the angle between the carbon fibers and the direction of the applied load during the mechanical characterization. The prepregs were cut in the shape of rectangular sheets of 250 mm × 135 mm. Afterwards, the prepreg plies were stacked into a stainless-steel mold to obtain a final thickness of 2.5 mm after compaction. Next, the mold with the prepregs was pre-heated at 100 °C in an oven for 15 min to reduce the viscosity of the resin and the thermal gradient in the system at the beginning of the curing process. Under these conditions, the mold and prepregs were heated at 15–20 °C/min at the beginning of the curing process, as determined by our measurements, depending on the target temperature. The laminates were isothermally cured at 110, 135, 160, and 200 °C for 15 or 30 min with a pressure of 8.3 MPa in a Bench Top Laboratory Manual Press, model 4128 (Carver, Inc., Wabash, IN, USA). Once the curing time of 15 or 30 min was completed, the laminates were cooled down to room temperature by keeping the same pressure in an alternate press with the same characteristics but without using temperature. Composite laminates (250 mm × 135 mm × 2.5 mm in size) were removed from the mold (Figure 1) and cut into rectangular specimens of 250 mm × 25 mm × 2.5 mm using a water jet cutting machine for subsequent tensile testing.

### 2.3. Differential Scanning Calorimetry

Kinetic parameters were obtained from DSC measurements in a double-furnace power compensation differential calorimeter (PerkinElmer DSC 8000, Waltham, MA, USA) with an intercooler. Prior to obtaining the thermograms, each sample of Aerotuf resin, cured at different temperatures and times, was pressed in an aluminum pan. Heating rates ranging from 1 to 20 °C/min were considered for non-isothermal measurements, and the baselines were obtained through a second run at the same heating rate. Residual reaction heat was obtained from samples processed at several curing temperatures (for either 15 or 30 min) through a dynamic scan at a heating rate of 10 °C/min. It is assumed that the pressure used during compression molding does not affect the curing reaction but the heating rate, in similar conditions to those reported in [36]. Sample weight, ranging from 5 to 10 mg, was determined before and after DSC measurements, and mass loss was not observed. Curing kinetics were obtained by considering the ASTM standards E2890 and E698 [37,38], which consider the Flynn–Wall–Ozawa and Kissinger methods, respectively. Alternatively, the Borchardt–Daniels [39] and Friedman [40] approaches were also considered. Here the isothermal characterization was performed at 130, 135, and 145 °C for kinetic model validation after its determination by the aforementioned methods.

### 2.4. Tensile Tests

Tensile measurements were performed on rectangular specimens according to the ASTM standard D3039 [41]. A Shimadzu universal/tensile testing machine (Shimadzu Corporation, Kyoto, Japan) was used for measurements. Tests were carried out at room temperature at a crosshead rate of 2 mm/min. At least five specimens were tested for each experimental condition.

## 3. Results and Discussion

### 3.1. Non-Isothermal Characterization by DSC: Kinetic Parameters

**Figure 2:**
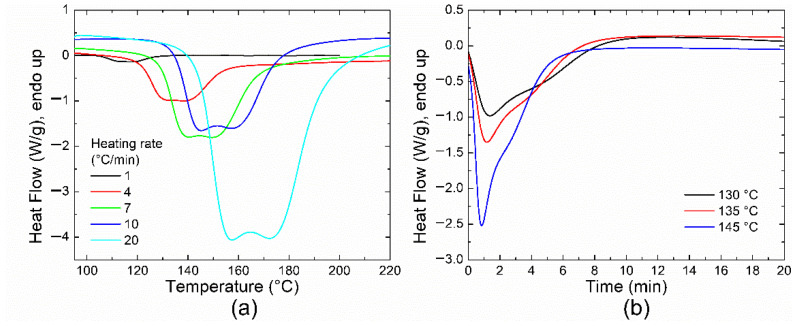
(**a**) DSC curves at different heating rates; (**b**) isothermal thermograms for the Aerotuf resin.

Usually, if more than one thermal process occurs during the curing process, two or more exothermic peaks or even shoulders can be easily observed in non-isothermal thermograms. In contrast, they are not well-resolved in the isothermal DSC curves [42]; thus, it is more convenient to obtain the kinetic parameters from dynamic measurements. The thermograms for the Aerotuf resin were obtained at five different heating rates *β* (Figure 2a). For each DSC curve, two exothermic peaks with similar intensities are clearly observed, associated with two polymerization stages; particularly, they are well defined at high *β* values and shift up to higher temperatures as the heating rate increases. For example, at *β* = 4 °C/min the first/second peak occurs at 130/140 °C, but when *β* is increased up to 20 °C/min this peak is shifted up to 157/172 °C. On the other hand, the isothermal DSC curves shown in Figure 2b illustrate the contribution of these two polymerization stages by the appearance of a peak and a shoulder. After these features, continuous diffusion-controlled reactions are observed as a flattened non-zero reaction tail.

On the other hand, kinetics parameters such as activation energy (*E_a_*) and pre-exponential factor (or Arrhenius frequency factor *Z*) can be obtained by considering the general rate equation:

(1) $$\frac{d\alpha }{dt}=k\left(T\right)f\left(\alpha \right)$$

where *dα*/*dt* is the reaction rate, which is assumed to be proportional to the measured heat flow *dH*/*dt*; *α* is a fractional conversion; *k*(*T*) is a specific rate constant at temperature *T*; and *f*(*α*) is determined by the cure mechanism. Typically, the most common functions are the nth-order (*f*(*α*) = (1 − *α*)*^n^*) and the autocatalytic (*f*(*α*) = *α^m^*(1 − *α*)*^n^*) reactions, where *m* and *n* are reaction orders.

Assuming Arrhenius behavior, *k*(*T*) is defined as:

(2) $$k\left(T\right)=Ze^{-\frac{Ea}{RT}}$$

where *Z* is the Arrhenius frequency factor, *E_a_* is the activation energy, and *R* is the gas constant.

**Figure 3:**
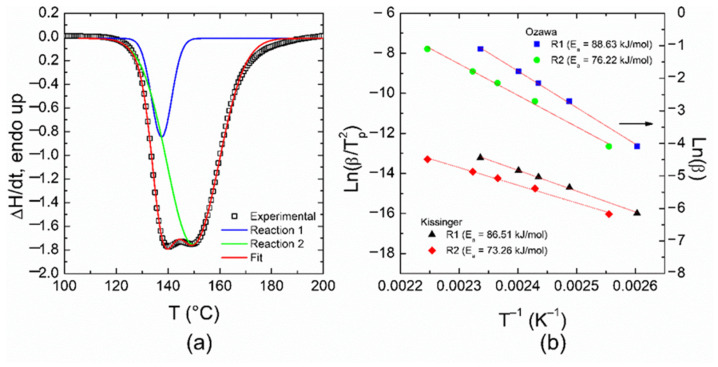
(**a**) Deconvolution of the DSC thermogram at *β* = 7 °C/min (after baseline correction) by using two Gaussian functions; (**b**) Ozawa and Kissinger plots for the determination of kinetic parameters. Fitting curves are shown as red lines.

From Figure 2, the measured DSC curve can be considered as the convolution of two different processes or reactions due to the existence of two exothermic peaks. This confirms the complex polymerization behavior during the curing process; one of these reactions may act as a catalyst for the other. Therefore, for the study of polymerization kinetics, each DSC curve was deconvoluted by using Gaussian curves. A good agreement with the experimental data is found, as shown in Figure 3a, although it is possible to use other mathematical models as previously reported in the literature (such as Pearson VII distribution, see Reference [43]).

Because both reactions start almost at the same temperature, the overlap of the deconvoluted curves does not allow the two peaks shown in the DSC curves to be analyzed using the method proposed by Martin et al. [44], since the test of consistency was not verified in this case; the two reactions are not consecutive (they are almost simultaneous). However, the first peak (at low temperature) can be undoubtedly associated with reaction 1, and the second one at high temperature to reaction 2, with different activation energies. These reactions can be correlated with heterocyclic/epoxy ring-opening and side reactions [25]. In addition, at this stage it is not possible to affirm whether both reactions are independent. After deconvolution, it is possible to obtain the reaction heat for each process involved in the total reaction, as listed in Table 1, for further determination of the kinetic parameters associated with such processes.

As shown in Table 1, the peak positions for both reactions are shifted up to higher temperature as the heating rate increases. As previously mentioned above and in a similar way, the corresponding total reaction heat (Δ*H*_Exp_) also increases (except for *β* = 10 °C/min). It is worth mentioning that the sum of the reaction heat Δ*H*_Peak1_ and Δ*H*_Peak2_, associated with the deconvoluted curves, is quite similar to the total experimental reaction heat Δ*H*_Exp_ since the ratio Δ*H*_Exp_/(Δ*H*_Peak1_ + Δ*H*_Peak2_) is close to 1. In accordance with Table 1, reaction 1 contributes between 15% and 24% to the total reaction and reaction 2 between 76% and 87% considering the heating rates of 1 to 20 °C/min. Due to the almost simultaneous occurrence of both reactions, reaction 1 could trigger the second one, which is possibly related to a catalyst included in the Aerotuf formulation. Based on its majority contribution to the total reaction, reaction 2 can be assigned tentatively to epoxy ring-opening/epoxy-amine addition. In our case, DSC cannot distinguish subsequent reactions (such as etherification).

**Figure 4:**
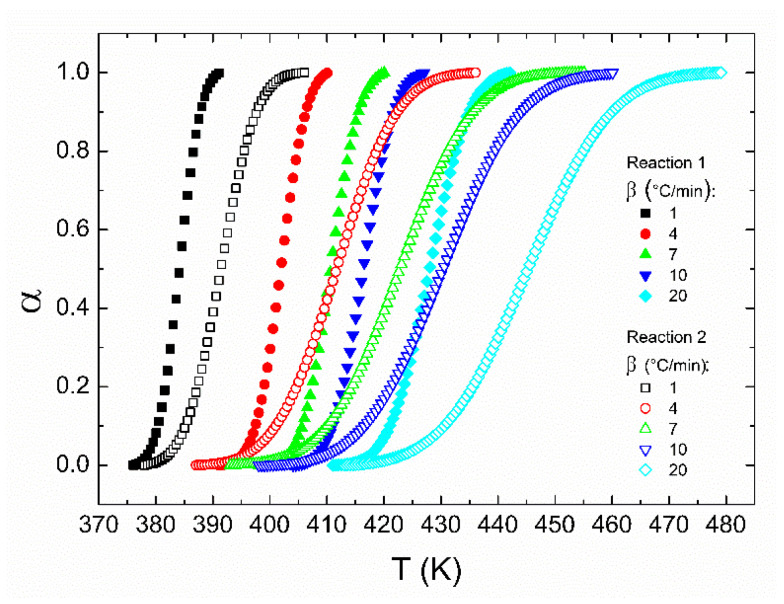
Non-isothermal degree of conversion as a function of both cure temperature and heating rate for the deconvoluted reactions. The relative weight of each reaction is not considered here.

Figure 3b shows the Kissinger and Ozawa plots for both reactions. Because the curing mechanism is unknown, it is convenient to use these approaches first. The Kissinger method considers the relationship between ln(*β/T_p_*^2^) and 1/*T_p_*, *T_p_* being the position of the exothermic peak. The average activation energy can be determined from the slope (=−*E_a_/R*) of the fitting line, within an error of around 5% [23]. The Ozawa method establishes that the resultant slope in the ln(*β*) vs. 1/*T_p_* plot is equivalent to −1.051 *E_a_/R*. These methods assume that at the peak temperature the degree of conversion is independent of the heating rate. From Figure 3b, by comparing the methods used in the calculations, the average activation energies obtained for each reaction are quite similar, and considering a particular approach, the difference between the *E_a_* values for the reactions is not so significant (~10 kJ/mol). This could explain why there is an overlap in the DSC thermograms. It is worth mentioning that the average activation energy (or energy barrier) for reaction 2 is lower than that for reaction 1, implying that the latter is more sensitive to the temperature [43]. The degree of cure α for each reaction can be obtained from the following relationship:

(3) $$\frac{1}{H_{T}}\frac{dH(t)}{dt}$$

where *H_T_* is the total heat of the reaction, and *H*(*t*) is the heat of the reaction up to the time *t*. Furthermore, notice that for the overall reaction, the total conversion degree must consider the relative contributions of each, as shown in the last two columns of Table 1. The degree of cure for both reactions is shown in Figure 4, where the overlap of the reactions is observed again.

**Figure 5:**
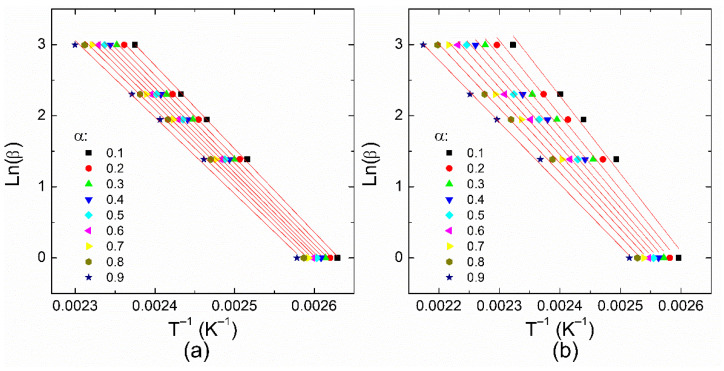
Flynn–Wall–Ozawa plots at different conversion degrees for (**a**) reaction 1 and (**b**) reaction 2. Fitting lines are also shown as red lines.

**Figure 6:**
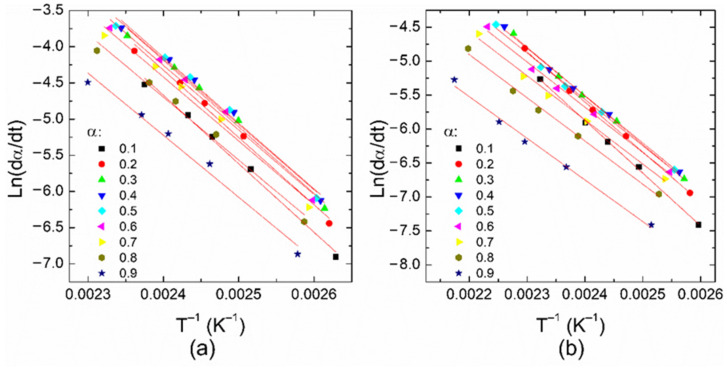
Friedman plots for (**a**) reaction 1 and (**b**) reaction 2 at different conversion degrees. Fitting curves are also shown as red lines.

From Figure 4, the “temperature window” for completing the reactions can be observed. For example, at 10 °C/min, reaction 1 is completed within a temperature window of ~25 K, whereas reaction 2 requires ~45 K. This is related to the differences in the average activation energies considering that reaction 1 is more sensitive to the temperature than reaction 2, as mentioned above. Thus, to obtain a complete polymerization process, the temperature window of reaction 2 can act as a limiting factor that must be taken into account during the manufacturing of solid elements. Based on the data of Figure 4, it is possible to build the Flynn–Wall–Ozawa and Friedman plots, as shown in Figure 5 and Figure 6, respectively. Therefore, the behavior of the apparent activation energy along the entire curing range can be understood through the iso-conversional methods.

**Figure 7:**
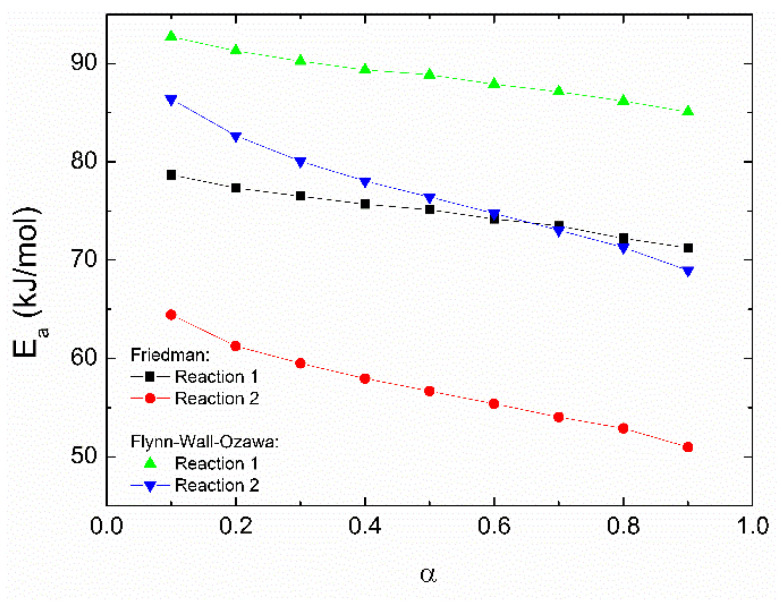
Apparent activation energy for the reactions 1 and 2 as a function of the conversion degree, from the Flynn–Wall–Ozawa and Friedman methods. Lines provide a visual guide.

Based on the Flynn–Wall–Ozawa method, *T_α_* defines the temperature that corresponds to the degree of cure *α*; the apparent activation energy can be obtained from the slope of the ln(*β*) vs. *Tα*^−1^ plot, which is equivalent to −1.051 *E_a_*/*R*. From the Friedman method, the slope in the ln(*dα/dt*) − *Tα*^−1^ plot provides the value of −*E_a_/R*, as illustrated in Figure 6. The *E_a_* values obtained from these methods are plotted in Figure 7 as a function of the conversion degree. From this figure, one can see that the activation energy values for a particular reaction are similar independently of the method considered in their calculation since the difference in the values for each reaction is around 10 kJ/mol. The apparent activation energy diminishes as the curing degree increases. This behavior has been attributed to the increasing number of hydroxyl groups in the propagation stage of an autocatalytic reaction; these –OH groups are generated as a consequence of the amines reacting with epoxides [21,28]. In the beginning, high activation energy or a high energy barrier can be caused by the steric hindrance during the epoxy-amine addition [45]. A further increase in *E_a_* values at high conversion degrees, associated with a possible diffusion process, is not observed.

**Figure 8:**
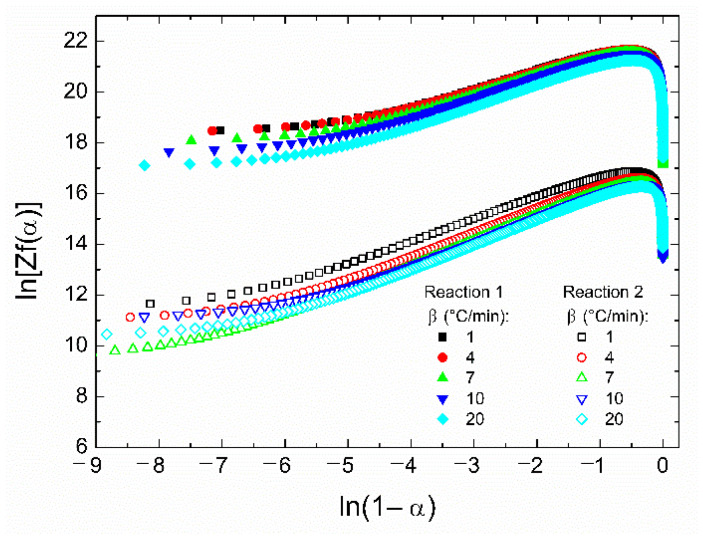
Plots of ln[*Zf*(*α*)] vs. ln(1 − *α*) for reactions 1 and 2 at several heating rates. The average activation energy obtained from the Ozawa method was used.

The activation energy values obtained from the Friedman and Flynn–Wall–Ozawa methods are close to those obtained from the Kissinger and Ozawa methods, previously discussed. Therefore, to get an adequate mathematical model for the total reaction, the average activation energy obtained with the Ozawa method was chosen for further calculations. If another activation energy value is used (such as that obtained from the Kissinger method), the following discussions do not significatively change. Figure 8 shows the ln[*Zf*(*α*)] vs. ln(1 − *α*) plots which are helpful for the determination of the reaction mechanism, since if the plot is a straight line, the polymerization follows nth-order kinetics; otherwise, it follows an autocatalytic process [43].

Since the activation energy of reaction 2 is lower than that of reaction 1, the set of curves are vertically shifted to lower values, and despite the differences in their *E_a_* values, the line shapes are similar. This means that the reactions follow similar polymerization kinetics. Moreover, there are two particular characteristics: the curves are not straight lines in the whole range of values of ln(1 − *α*), and the maximum values are around *α* = 0.3 (or equivalently, ln(1 − *α*) ≈ −0.36). Although many epoxy resins follow nth-order reactions (with maximum values at *α* < 0.20), this is not the case. In the plot, the region between *α* = 0.3 and 0.8 is linear; however, at higher conversion degrees it does not occur. This strongly suggests that the polymerization process is autocatalytic for both reactions [43].

The Borchardt–Daniels approach was used for the determination of the other kinetic parameters. According to this method, the reaction models are rewritten, for the nth-order reactions as:

(4) $$\ln(\frac{d\alpha }{dt})=\ln(Z)-\frac{E_{a}}{RT}+n\ln(1-\alpha )$$

for autocatalytic reactions as

(5) $$\ln(\frac{d\alpha }{dt})=\ln(Z)-\frac{E_{a}}{RT}+n\ln(1-\alpha )+m\ln(\alpha )$$

These equations can be solved by multiple linear regression methods. Here, the average activation energy obtained from the Ozawa method was used to reduce the parameters to be determined during the linear regression procedure. Although the autocatalytic nature of the reactions was determined from the plots of ln[*Zf*(*α*)] vs. ln(1 − *α*), the nth-order reaction was also considered. In this case, the obtained *n* and *Z* values provide a coefficient of determination (*R*^2^) ranging from 0.34 to 0.69. On the other hand, the kinetic parameters for the autocatalytic reactions provide an *R*^2^ value very close to 1, as shown in Table 2, which confirms that the curing reactions of this resin system are autocatalytic in nature.

Notice from Table 2 that the *n* value is close to 1 and slightly increases as the heating rate is increased, whereas the behavior of the *m* values is the opposite, in such a manner that the sum *m* + *n* is ~1.5. This means that the proposed mechanism for the curing reaction is independent of the heating rates [46]. The pre-exponential factor *Z* also diminishes as the heating rate is increased; the *Z* value for reaction 1 is two orders of magnitude greater than that for reaction 2. Regarding the *m* values for reaction 1, they are also greater than those for reaction 2. These results confirm that reaction 1 is faster than reaction 2.

**Figure 9:**
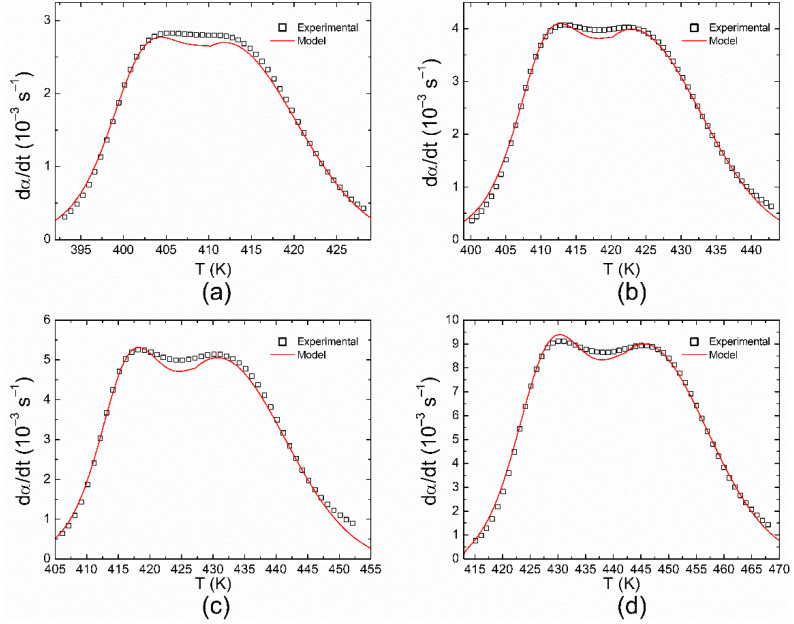
Conversion rate as a function of temperature at different heating rates: (**a**) 4; (**b**) 7; (**c**) 10; and (**d**) 20 °C/min.

**Figure 10:**
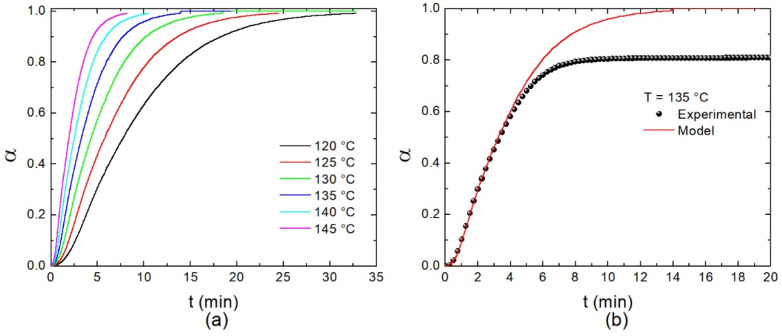
(**a**) Calculated isothermal thermograms by considering the autocatalytic model and the kinetic parameters of Table 2. For comparison, experimental results are shown in (**b**).

Once the kinetic parameters were obtained from the autocatalytic model, a good agreement with the experimental results was observed considering the relative weight of each reaction in the total process, as listed in the last two columns of Table 1. Figure 9 shows the conversion rate as a function of the temperature at 4, 7, 10, and 20 °C/min. Notice that the model presents small deviations from the experimental results for values located between the exothermic peaks. This could be related to the method used (Gaussian curves) during the deconvolution process. However, as shown in Figure 10, it does not considerably affect the determination of the isothermal curves with the autocatalytic model, and therefore other curves were not considered for the deconvolution.

The theoretical isothermal curves at six different temperatures are shown in Figure 10a. These curves were calculated from the whole set of results obtained by DSC. A good agreement with the experimental results is confirmed with the autocatalytic model previously discussed. However, a difference is observed in the ultimate conversion; in fact, at 135 °C the ultimate *α* value is around 0.8, whereas the model predicts that complete conversion is possible, as shown in Figure 10b. This evidences the presence of cured resin and unreacted residues in the material, and thus, the final stage in the cross-linking process is controlled by diffusion. As the curing temperature increases, the ultimate curing degree also increases and is determined by the residual heat.

### 3.2. Residual Conversion Degree

**Figure 11:**
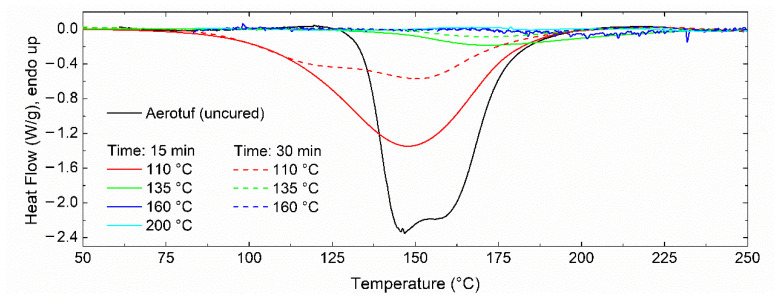
Non-isothermal DSC curves of the Aerotuf resin at different curing times and temperatures. Heating rate: 10 °C/min.

Thermograms for uncured and cured samples at several temperatures and two different times (15 and 30 min) were measured for the determination of residual reaction heat (see Figure 11 and Table 3). The temperatures were chosen by considering Figure 2 and *β* = 10 °C/min: 110 °C is lower than the onset of the exothermic peak (~135 °C), 160 °C matches one of the maximum peaks, and 200 °C is the temperature after curing completion. Most of the curves in Figure 11 show only one exothermic peak; however, at 110 °C and for a curing time of 30 min, it is possible to observe two peaks with different intensities. The exothermic peak shifts up to higher temperatures as the curing temperature or curing time increases. This behavior agrees with that stated above: Reaction 1 is faster than reaction 2; therefore, at low temperatures the two reactions are incomplete, and at high temperatures, it is expected that only reaction 1 is close to its completion (or it is complete). In other words, based on the relative contribution of each reaction to the total curing process, as shown in Table 1, the narrow temperature window needed for reaction 1 could be related to the additive (or modifier) consumption included in the modified epoxy resin, whereas the second exotherm includes the contribution of the oxirane ring-opening and subsequent reactions [28]. Thus, a wider temperature window is required to attain a complete degree of curing.

**Figure 12:**
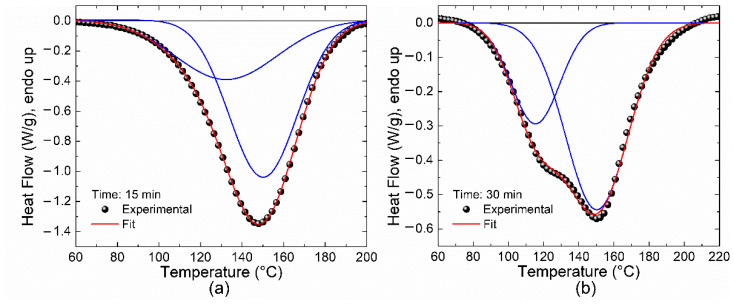
Non-isothermal thermograms at 10 °C/min for the Aerotuf resin cured at 110 °C for (**a**) 15 min; (**b**) 30 min. Deconvoluted curves are also shown as solid lines.

Based on the results listed in Table 3, at a certain time both the enthalpy and the residual conversion degree drop as a function of the curing temperature. The resin is almost completely cured for temperatures higher than 160 °C and a curing time of 15 min or, when a time of 30 min has elapsed, for temperatures higher than 135 °C. It is worth noting that at 135 °C and 15 min, the residual conversion degree is around 0.14, which is close to the value previously shown in Figure 10b. On the other hand, DSC curves were recorded from the samples listed in Table 3; they were deconvoluted to follow their evolution as a function of temperature and time. Only the curves for resins cured at 110 °C presented two exothermic peaks, as illustrated in Figure 12, whereas the samples under other conditions exhibited only one peak or none, depending on their conversion degree. This means that at a temperature of 110 °C, two reactions must be performed to complete the curing process, whereas at temperatures equal to or higher than 135 °C there is only one reaction required. Thus, it is expected that the mechanical properties of the resin, and hence the performance of the [±45°] WCFF/Aerotuf composite laminates, can be compromised because of the incomplete curing reaction.

### 3.3. Influence of the Curing Temperature on the Mechanical Properties

**Figure 13:**
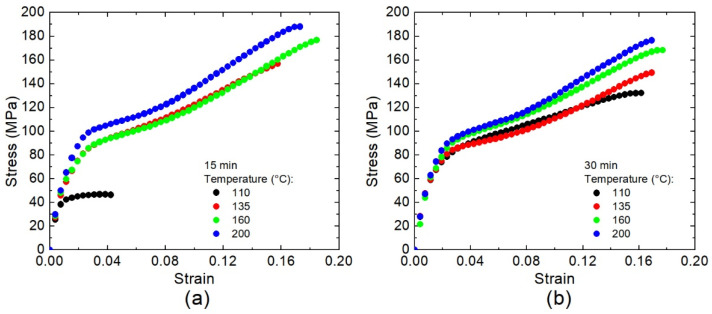
Stress–strain for [±45°] WCFF/Aerotuf composite laminates molded for (**a**) 15 min; (**b**) 30 min, at several curing temperatures.

**Figure 14:**
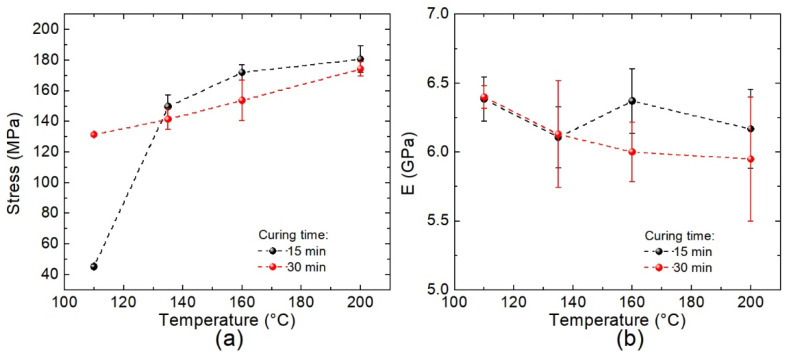
(**a**) Ultimate tensile strength; (**b**) Young’s modulus for [±45°] WCFF/Aerotuf composite laminates manufactured at several curing temperatures and two molding times.

There are several alternatives for the curing process of [±45°] WCFF/Aerotuf materials. According to the previous results obtained via DSC, the simplest is to maintain the prepregs at a fixed temperature (isothermal curing) and pressure during a certain time. This approach was chosen in order to correlate the mechanical properties of [±45°] WCFF/Aerotuf laminates with the kinetic models discussed above. Distinct molding pressures did not provide important differences in the mechanical properties; thus, arbitrarily, a load of 8.3 MPa in the tensile testing machine was used. Average stress–strain curves shown in Figure 13 exhibit the elastic and plastic regions, which are typical for polymer materials. In this case, the applied load is transferred through the resin and the carbon fibers in the elastic region at low strains. On the other hand, in the plastic region, the thermoset resin is mainly responsible for the load transfer until the failure occurs by plastic deformation. One can see from Figure 13a that at 15 min of curing time, the plots form two groups: the curve related to materials cured at 110 °C and others. At the elapsed time of 30 min, the situation is different since only one group is observed in Figure 13b. These results are consistent with those obtained via DSC (see Table 3) since, at 110 °C, the attained degree of curing is ~0.11, whereas *α* > 0.85 for the other temperatures considered in this study. Therefore, average values of the ultimate tensile strength (UTS) and Young’s modulus of the [±45°] WCFF/Aerotuf composites as a function of the temperature and curing time can be found, as shown in Figure 14.

Notice from Figure 14a that the UTS increases monotonically as a function of temperature regardless of the curing time (15 or 30 min), reaching higher values at 15 min of molding time (except for a molding temperature of 110 °C, which is lower than the onset of the exothermic process observed by DSC for *β* > 4 °C/min). It seems that the UTS increases asymptotically up to ~180 MPa. This behavior can be related to the curing degree discussed above; i.e., *α*~1 at the higher temperatures considered in this study. The UTS values at 110, 135, and 160 °C are approximately 25%, 83%, and 95%, respectively, of the obtained maximum value at 200 °C. These UTS values match the residual conversion degrees shown in Table 3 since the curing degrees attained at 110, 135, 160, and 200 °C are 0.11, 0.86, 0.97, and 1, respectively, for a curing time of 15 min. Following the same reasoning for laminates cured for 30 min, the UTS values are 75%, 81%, 88%, and 100% when the curing temperatures are 110, 135, 160, and 200 °C, respectively. However, from Table 3, the conversion degrees are, respectively, 0.53, 0.96, 0.99, and 1, which does not follow the same trends as observed for 15 min of curing time. Because of the asymptotic behavior, a significant increase in the UTS values is not expected at temperatures higher than 200 °C. The specific surface area of the carbon fiber or the functional groups attached to their surface could influence the curing process in a similar manner to that previously reported in [47]; thus, the mechanical properties change with time due to different molecular interactions between the cured resin and the carbon fibers. This might explain the discrepancies observed for these two curing times.

Finally, the values of Young’s modulus shown in Figure 14b are almost independent of both the curing time and the molding temperature (within the experimental error). As observed, Young’s modulus ranges from 5.9 ± 0.4 to 6.4 ± 0.2 MPa due to the changes in the processing conditions. Such differences appear to be insignificant taking into account the experimental error obtained from measurements; thus, it would be difficult to establish any behavioral tendency for Young’s modulus of the produced laminates. Nonetheless, we could infer that the curing degree obtained for each produced composites strongly impacts the plastic region where the load is transferred from the resin to the carbon fibers; this probably causes the elastic region to remain unaffected, leading to values relatively constant in our material Young’s modulus values. However, we consider that a more in-depth analysis of the evolution of thermo-elastic properties of the epoxy resin and carbon fibers throughout the curing process is needed, considering factors such as cure-induced chemical and thermal shrinkages, which contribute to the development of stress and residual deformation in thermosetting composite laminates [48,49].

## 4. Conclusions

Aerotuf, a modified epoxy resin designed for aerospace applications was used in carbon fiber prepregs, and their kinetic parameters were determined by DSC measurements. Two exothermal peaks were observed in the thermograms, which were deconvoluted into two reactions. The analysis was performed using the Ozawa, Kissinger, Friedman, and Flynn–Wall–Ozawa approaches as well as the Borchardt–Daniels method. Our findings show that both reactions follow the autocatalytic model since good agreement with experimental data was obtained. With the kinetic parameters, we have identified the possibility of designing several curing procedures for the manufacturing of solid materials. Here, we used the simplest one (isothermal) in the manufacturing of [±45°] WCFF/Aerotuf composite laminates. At low temperatures (110 °C in our case) the curing time is relevant because the composite material is not fully polymerized since the residual conversion degree goes from ~0.89 to ~0.47 when time varies from 15 to 30 min. Therefore, the optimal temperature and time can be selected depending on the energy cost (related to temperature) and time. Some alternatives are 160 °C for 15 min and 135 °C for 30 min, which lead to a residual conversion degree of ~0.04, which can be considered as sufficient for many applications. This is consistent with that reported by Ramírez-Herrera et al. in [28] using the FTIR technique. However, if the maximum values for UTS and Young’s modulus are of interest, a curing time of 160 °C for 15 min could be sufficient. Furthermore, we identified a close relationship between conversion degree and the UTS values obtained from the tensile tests, whereas Young’s modulus remains almost unaffected.

This article sheds new light on understanding the influence that epoxy resin curing kinetics have on the development carbon fiber composites with enhanced physical and mechanical properties to produce lightweight aerospace components.

## Figures and Tables

**Table 1:** Peak positions and enthalpies for deconvoluted DSC curves.

Heating Rate (°C/min)	*T*_Peak__1_ (K)	*T*_Peak__2_ (K)	Δ*H*_Peak__1_ (J/g)	Δ*H*_Peak__2_ (J/g)	Δ*H*_Exp_ (J/g)	$\frac{\Delta H_{Exp}}{\Delta H_{Peak1}+\Delta H_{Peak2}}$	Relative Weight to the Total Reaction	
							Reaction 1	Reaction 2
1	384	391	30.43	98.74	126.06	0.98	0.24	0.76
4	402	412	46.32	264.92	304.29	0.99	0.15	0.87
7	411	423	69.34	373.55	435.45	0.98	0.16	0.86
10	417	431	67.73	316.39	385.84	1	0.18	0.82
20	428	445	106.50	348.78	449.27	0.99	0.24	0.78

**Table 2:** Kinetic parameters for the Aerotuf resin, by considering the autocatalytic model. The average activation energy was obtained by the Ozawa method.

Reaction (*E_a_*, kJ/mol)	*β* (°C/min)	*m*	*n*	*Z* (s^−1^)	*R* ^2^
1 (88.63)	1	0.65	0.89	7.40 × 10^9^	0.9995
	4	0.65	0.90	7.41 × 10^9^	0.9993
	7	0.63	0.91	6.58 × 10^9^	0.9994
	10	0.62	0.92	6.06 × 10^9^	0.9995
	20	0.58	0.95	4.63 × 10^9^	0.9992
2 (76.22)	1	0.60	0.96	5.96 × 10^7^	0.9997
	4	0.48	1.03	4.23 × 10^7^	0.9984
	7	0.47	1.08	3.58 × 10^7^	0.9992
	10	0.45	1.08	3.18 × 10^7^	0.9989
	20	0.45	1.09	2.93 × 10^7^	0.9991

**Table 3:** Calculated values for resin samples cured at different times and temperatures.

Curing Time (min)	Curing Temperature (°C)	*T_Peak_* (°C)	Enthalpy (J/g)	Conversion Degree (Residual)
0	Uncured	-	445.40	1.000
15	110	147	397.46	0.892
	135	172	62.08	0.139
	160	232	14.32	0.032
30	110	151	207.13	0.465
	135	172	19.18	0.043
	160	189	2.98	0.007

## Data Availability

Not applicable.
